# Prognostic Value of Neutrophil-to-Lymphocyte Ratio in Localized and Advanced Prostate Cancer: A Systematic Review and Meta-Analysis

**DOI:** 10.1371/journal.pone.0153981

**Published:** 2016-04-20

**Authors:** Lu Tang, Xintao Li, Baojun Wang, Guoxiong Luo, Liangyou Gu, Luyao Chen, Kan Liu, Yu Gao, Xu Zhang

**Affiliations:** State Key Laboratory of Kidney Disease, Department of Urology, Chinese PLA Medical Academy, Chinese People’s Liberation Army General Hospital, Beijing, 100853, China; The First Affiliated Hospital of Nanjing Medical University, CHINA

## Abstract

**Objective and Background:**

Increasing evidence suggests that inflammation plays an essential role in cancer development and progression. The inflammation marker neutrophil–lymphocyte ratio (NLR) is correlated with prognosis across a wide variety of tumor types, but its prognostic value in prostate cancer (PCa) remains controversial. In the present meta-analysis, the prognostic value of NLR in PCa patients is investigated.

**Methods:**

We performed a meta-analysis to determine the predictive value of NLR for overall survival (OS), recurrence-free survival (RFS), and clinical features in patients with PCa. We systematically searched PubMed, ISI Web of Science, and Embase for relevant studies published up to October 2015.

**Results:**

A total of 9418 patients from 18 studies were included in the meta-analysis. Elevated pretreatment NLR predicted poor OS (HR 1.628, 95% CI 1.410–1.879) and RFS (HR 1.357, 95% CI 1.126–1.636) in all patients with PCa. However, NLR was insignificantly associated with OS in the subgroup of patients with localized PCa (HR 1.439, 95% CI 0.753–2.75). Increased NLR was also significantly correlated with lymph node involvement (OR 1.616, 95% CI 1.167–2.239) but not with pathological stage (OR 0.827, 95% CI 0.637–1.074) or Gleason score (OR 0.761, 95% CI 0.555–1.044).

**Conclusions:**

The present meta-analysis indicated that NLR could predict the prognosis for patients with locally advanced or castration-resistant PCa. Patients with higher NLR are more likely to have poorer prognosis than those with lower NLR.

## Introduction

Prostate cancer (PCa) is the most common malignancy in men and a leading cause of cancer-related death [1]. The incidence of PCa has increased markedly in recent years. Despite significant improvements in diagnostic and therapeutic strategies, the prognosis of PCa remains poor, especially in patients with metastatic castration-resistant PCa (mCRPC) [2]. Thus, finding prognostic markers for PCa is urgently needed to help deliver personalized measures and thus prevent and treat the disease at an early stage.

Increasing evidence suggests that inflammation plays an essential role in cancer development and progression [3, 4] and that systemic inflammation is associated with poor prognosis in a number of cancers [5, 6]. Increased levels of pro-inflammatory cytokines in cancer patients may reflect both disease activity and the innate response of the host to the tumor [7]. Cancer-related inflammation affects the tumor microenvironment [8]. Moreover, inflammatory cells have significant effects on tumor development; thus, systemic inflammation markers may represent useful prognostic biomarkers [9, 10]. Neutrophil–lymphocyte ratio (NLR) is an indicator of general immune response to various stress stimuli, and it is correlated with prognosis across a wide variety of tumor types. High NLR is associated with adverse outcomes in a variety of malignancies [11, 12].

Several retrospective studies have evaluated the prognostic significance of baseline NLR in patients with PCa. Several studies demonstrated that NLR had prognostic value in localized and advanced PCa [13–17]; on the contrary, other studies reported that high serum NLR had no prognostic value in patients with PCa [17, 18]. Thus, the prognostic value of NLR in PCa remains controversial. A previous meta-analysis on NLR in patients with urologic tumors found that NLR could predict overall survival (OS) and recurrence-free survival (RFS) in PCa [19]. However, this meta-analysis included only six original studies, all of which involved patients with CRPC. Increasing studies on NLR and PCa have been reported recently. Thus, we performed a systematic review and meta-analysis of published studies to determine the predictive value of NLR for PCa prognosis and clinical features.

## Methods

### Search strategy

A systematic literature search was performed using the electronic databases PubMed, ISI Web of Science, and Embase up to October 2015. Search terms included “NLR,” “neutrophil to lymphocyte ratio,” “prostate,” and “tumor, cancer, neoplasm or carcinoma.” The titles and abstracts of potential references were scanned carefully to exclude irrelevant articles. The remaining articles were evaluated to identify research that contained the topic of interest, and full texts were then reviewed comprehensively.

### Selection criteria

The studies included in this meta-analysis were randomized controlled studies or observational studies (case–control or cohort) that evaluated the association between pretreatment NLR and PCa prognosis. Studies were included if they (1) included patients histopathologically diagnosed with PCa, (2) provided pretreatment NLR and reported cut-off values, (3) focused on the prognosis of PCa, and (4) analyzed the associations between pretreatment NLR and survival outcomes (clinical RFS [CRFS], biochemical RFS [BRFS], and OS). Exclusion criteria were (1) non-English papers; (2) review articles, editorial comments, letters, expert opinion, conference abstracts, or case reports; (3) overlapping or duplicate data; (4) focus on animal models or cancer cells; (5) insufficient data for estimating hazard ratios (HRs) and 95% confidence intervals (CIs); or (6) full text unavailable.

All evaluations were performed independently by two reviewers to assure accurate inclusion of studies. When several studies contained overlapping data, the study with the largest data set was used. Multivariate outcomes were preferred to univariate outcomes if both were provided. However, univariate outcomes were acceptable if no multivariate results were presented. The given survival or mortality curves were used to calculate the values. If insufficient data were provided in the text, then we would contact the authors for the additional necessary data.

### Data extraction

All data were extracted by two independent reviewers. Disagreements in data extraction were resolved through consensus. The qualities of the included studies were assessed according to the Newcastle–Ottawa Quality Assessment Scale. This scale evaluates three aspects, namely, selection, comparability, and outcomes in the case and control groups. Studies with scores ≥6 were defined as high-quality studies [20]. The following relevant data were extracted in a predefined table: author, year, country, age, patient sample, follow-up duration, cut-off score, NLR (high/low), treatment, and endpoint (OS, CRFS, and BRFS).

Some studies presented survival data using the Kaplan–Meier curves, whereas we used GetData Graph Digitizer 2.26 (http://getdata-graph-digitizer.com/) to digitize and extract the relevant survival data.

The cut-off value for NLR varied among the included studies. Thus, we defined the NLR standard in accordance with the standards indicated in the original article. Several studies followed up patients for either CRFS or BRFS, and we analyzed these outcomes separately first and then together in the subgroup analysis.

### Statistical analysis

This meta-analysis was performed using Stata version 12.0 (StataCorp LP, TX, USA), and the statistical analysis was conducted according to the guidelines proposed by the Meta-analysis of Observational Studies in Epidemiology group. Associations between NLR and outcomes were reported as HRs and 95% CIs, either obtained directly from individual articles or calculated from indirect data. For the analysis of the relationship between NLR and clinicopathological features, odd ratios (ORs) and 95% CIs were synthesized as the effective value. Heterogeneity among studies was measured using *Q* and *I*^*2*^ tests. A fixed-effect model was used in the absence of significant heterogeneity; otherwise, a random-effect model was used. Potential publication bias was identified by Begg’s and Egger’s tests. The influence of publication bias on the overall effect was assessed by the “trim and fill” method described by Duval et al. [21]. *P* < 0.05 was considered statistically significant. All *P* values were two tailed.

Subgroup analyses were performed to investigate the associations of NLR with clinical features and prognosis in relation to geographic area, statistical methods, patient conditions, sample size, NLR cut-off value (3< and ≥3), follow-up duration, and recurrence type. The prognostic value of NLR value in terms of pathological stage, Gleason score, and lymph node involvement was only investigated in three, four, and two studies, respectively. Accordingly, we only performed pooled analysis on these factors. Furthermore, a sensitivity analysis was performed to examine the robustness of the pooled results. The meta-analysis followed the standard PRISMA checklist (S1 Table).

## Results

### Search results

**Fig 1:**
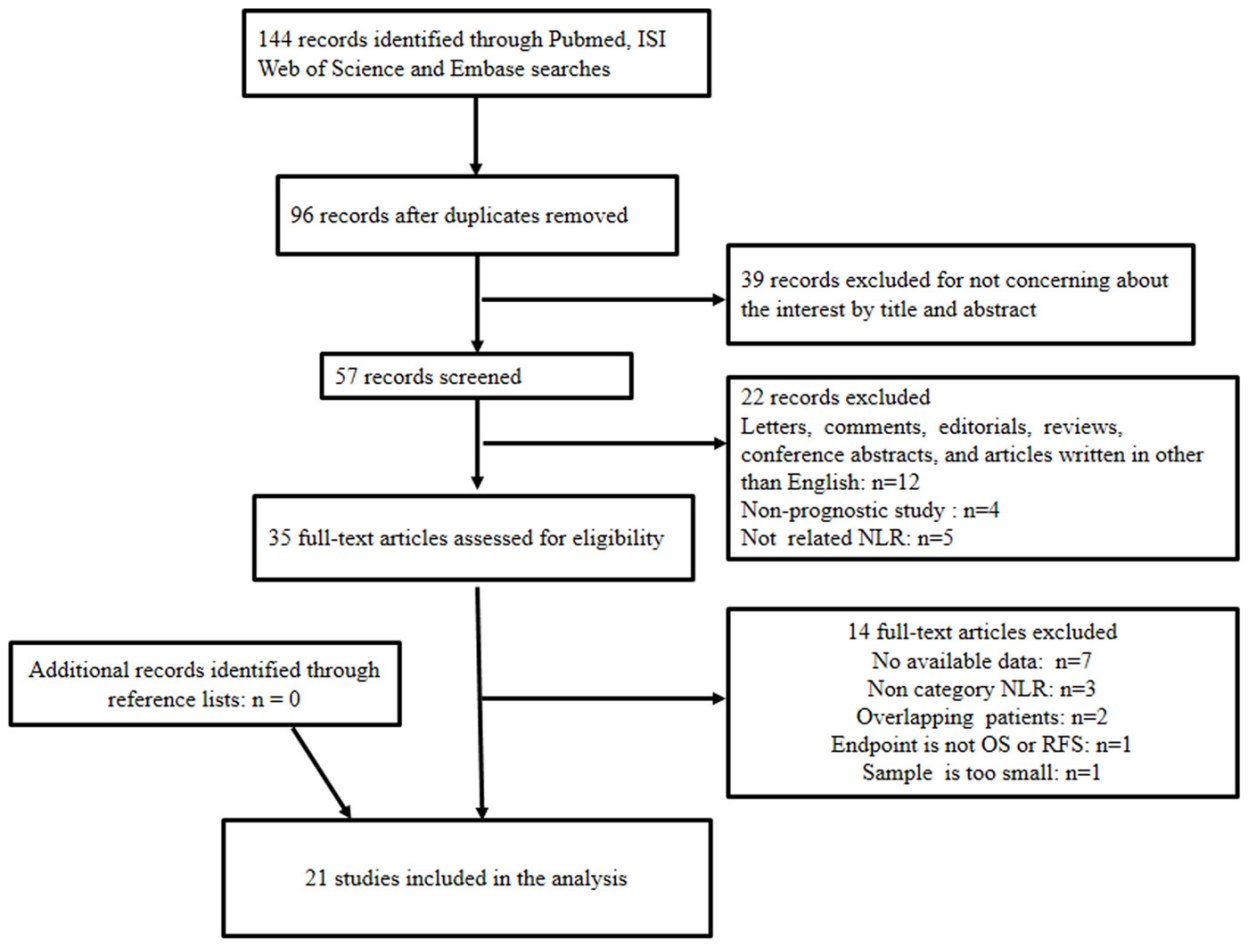
Flowchart of selection of studies for inclusion in meta-analysis.

A flowchart for the selection of literature is shown in Fig 1. A total of 144 records were returned using the search strategy; 96 of these records were retrieved after the exclusion of duplicated data. About 39 records were excluded after the initial evaluation of the title and abstract. Among the remaining 57 records, 23 were excluded including 12 that were letters, comments, editorials, reviews, and conference abstracts; 2 articles not written in English; 5 non-prognostic studies; and 4 studies unrelated to NLR. The full texts of the remaining 34 articles were assessed.

A total of 16 full-text articles were excluded, including 9 without available data, 3 without NLR category, 2 that enrolled overlapping patients, 1 with endpoints other than OS or RFS, and 1 with a sample size that was very small. Finally, 18 studies with 9418 patients were included in the meta-analysis. All the remaining 18 studies evaluated the prognostic value of pretreatment NLR in PCa and were reported within 5 years.

### Characteristics of eligible studies

Detailed information on the 18 studies is listed in S2–S4 Tables. These studies included 3 from the United States, 2 from Japan, 2 from Canada, 1 from the Netherlands, 1 from Italy, 2 from China, 1 from a European cohort, 1 from Korea, 1 from Turkey, 1 from a multinational cohort, 1 from the United Kingdom, 1 from Australia, and 1 from Switzerland. These studies included 9418 patients, with a median of 389 patients (range 33–1367) per study. Only 1 study included 2 cohorts, both of which focused on the prognostic role of pretreatment NLR in patients with mCRPC. The 17 other studies were retrospective observational cohort studies. Among all the included studies, 10 included locally advanced or mCRPC patients, and the remaining 8 included patients with localized PCa. All the NLR values were detected before treatment. Patients in 9 studies received docetaxel chemotherapy, patients in 4 received radical prostatectomy, patients in 3 received curative radiotherapy, and patients in 1 received chemotherapy with cabazitaxel or mitoxantrone. The treatment schedule was unavailable for 1 study. The median follow-up time was 18 months (range 0–156 months). The relationship between NLR and OS was investigated in 13 cohorts (11 by multivariate analysis), and 10 studies investigated the relationship between NLR and RFS (4 by multivariate analysis). Among the 10 studies analyzing NLR and RFS, 5 investigated BRFS and the 5 other investigated clinical CRFS. A total of 3 studies investigated the associations between NLR and pathological stage and Gleason score, and 2 investigated the relationship between NLR and lymph node involvement.

### Impact of NLR on recurrence in patients with PCa

**Fig 2:**
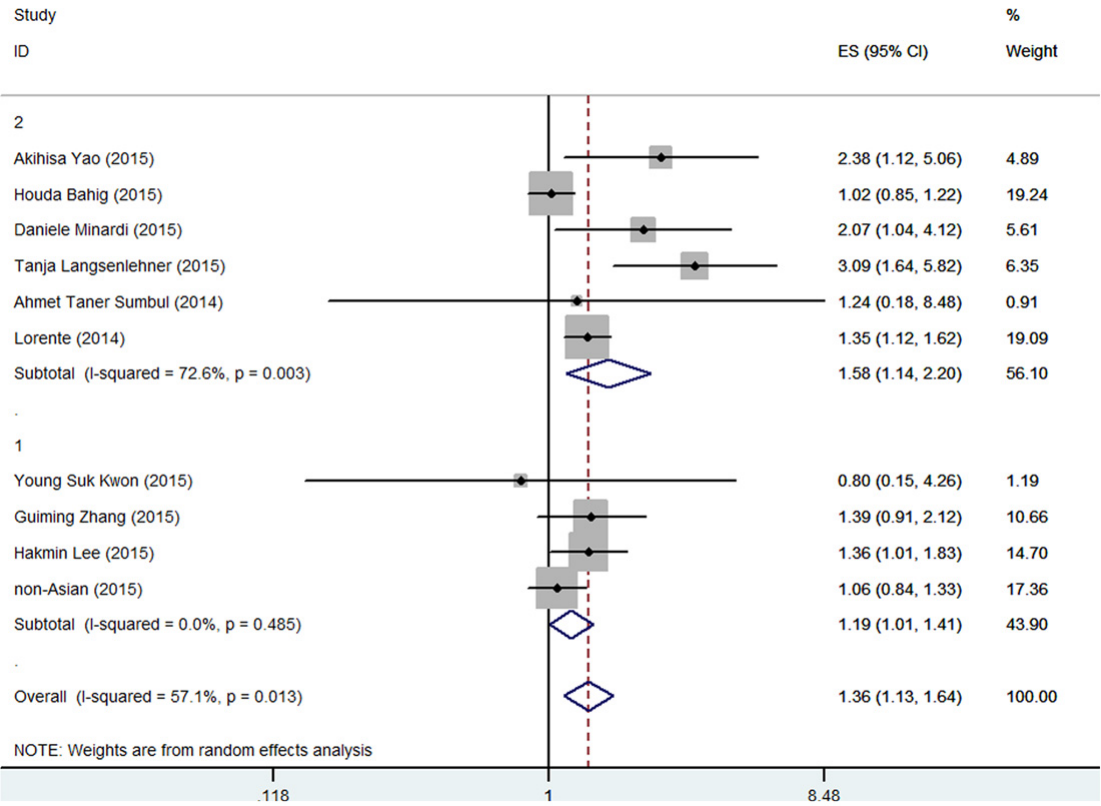
Meta-analysis of NLR value and RFS in PCa patients.

A total of 10 studies involving 4819 patients investigated the association between NLR and PCa recurrence. Patients with high pretreatment NLR had a significantly higher recurrence risk than those with low NLR (HR 1.367, 95% CI 1.126–1.636) (Fig 2 and Table 1). Significant heterogeneity was found among these studies (*I*^*2*^ = 57.1, *P* = 0.013), and thus the data were analyzed using a random-effect model.

**Table 1:** Results of the meta-analysis on predictive value of NLR in PCa.

	Overall survival				Recurrence free survival			
	N	HR	LCI	UCI	N	HR	LCI	UCI
**Overall**	13	1.628	1.41	1.879	10	1.357	1.126	1.636
**Geographic area**								
1. Asian	4	2.49	1.838	3.873	3	1.441	1.034	1.678
2. non-Asian	9	1.481	1.301	1.686	7	1.317	1.034	1.678
**Statistical methods**								
1. univariate	9	1.839	1.467	2.304	10	1.541	1.182	2.008
2. multivariate	11	1.73	1.483	2.018	4	1.577	1.269	1.961
**Patient**								
1. localized	2	1.439	0.753	2.75	7	1.334	1.051	1.693
2. locally advanced or mCRPC	10	1.507	1.395	1.627	3	1.393	1.165	1.665
**Sample size**								
1. <800	9	1.801	1.587	2.043	8	1.498	1.168	1.921
2. > = 800	4	1.326	1.143	1.539	2	1.102	0.944	1.286
**NLR standard**								
1. <3	3	1.365	1.22	1.527	4	1.191	1.008	1.405
2. > = 3	10	1.773	1.449	2.17	6	1.584	1.142	2.196
**Follow-up**								
1. <18	3	1.618	1.386	1.889	1	1.35	1.222	1.624
2. > = 18	4	1.959	1.1	3.486	7	1.334	1.051	1.693
**Recurrence type**								
1. BCR	NA	NA	NA	NA	5	1.208	1.078	1.354
2. clinical recurrence	NA	NA	NA	NA	5	1.84	1.064	3.184

NLR, neutrophil-to-lymphocyte ratio; mCRPC, metastatic castration-resistant prostate cancer; BCR, biochemical recurrence; LCI, lower confidence interval; UCI, upper confidence interval; HR, hazard ratio

### Effect of NLR on OS in patients with PCa

**Fig 3:**
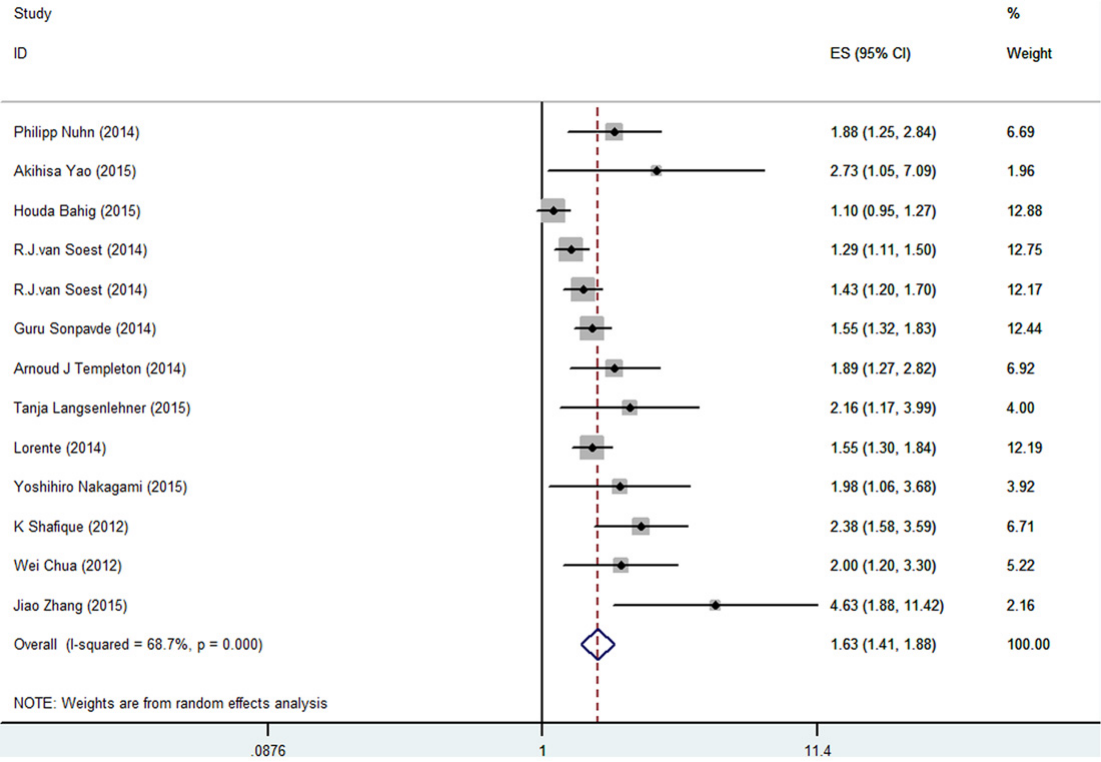
Meta-analysis of NLR value and OS in PCa patients.

The association between NLR and OS was evaluated in 13 cohorts including 6776 patients. Those with high pretreatment NLR had significantly poorer OS than those with low NLR (HR 1.628, 95% CI 1.41–1.879) (Fig 3 and Table 1). Significant heterogeneity was observed among these studies (*I*^*2*^ = 68.7%, *P* < 0.001).

We explored the heterogeneity by conducting subgroup analyses and meta-regression (S5 Table). In the subgroup analysis of RFS, no significant difference was found between high and low pretreatment NLR values in terms of RFS in sample sizes ≥800 (HR 1.102, 95% CI 0.944–1.286) (S1 and S2 Figs). However, the other subgroup analyses demonstrated better RFS in patients with low NLR. In the subgroup analyses of OS, no significant difference was observed between the high and low pretreatment NLR groups in patients with localized PCa (HR 1.439, 95% CI 0.753–2.75); however, higher pretreatment NLR indicated a poorer prognosis in patients with advanced PCa (HR 1.507, 95% CI 1.395–1.627) (S3 and S4 Figs). The predictive values of NLR were significant in all the other subgroups analyzed (Supplemental data). The meta-regression analysis suggested that geographic area (*P* = 0.017), patient type (*P* = 0.177), and sample size (*P* = 0.008) could partially explain the source of the heterogeneity of pooled OS. However, meta-regression analysis is only suitable for analyzing more than 10 studies, and we did not apply this method to the RFS pooled analysis.

### Correlations between NLR and clinicopathological features

The correlations between NLR and clinical features of PCa are presented in S6 Table. Three, four, and two studies were available for the pooled analysis with regard to pathological stage, Gleason score, and lymph node involvement, respectively. NLR was positively related to lymph node involvement (OR 1.616, 95% CI 1.167–2.139) but showed no significant correlation with pathological stage (OR 0.827, 95% CI 0.637–1.074) or Gleason score (OR 0.761, 95% CI 0.555–1.044). The combined OR value was calculated using the random-effect model because of the heterogeneity among the studies.

### Publication bias analysis

**Fig 4:**
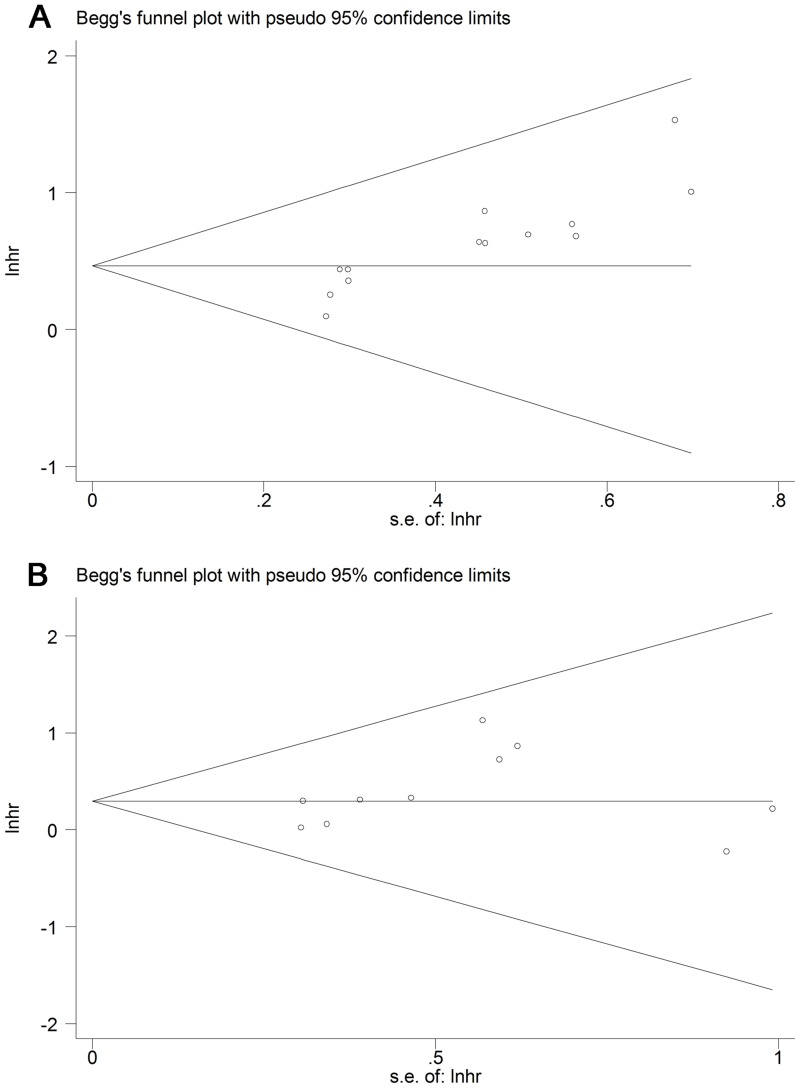
Begg’s test results of OS and RFS of PCa patients.

**Fig 5:**
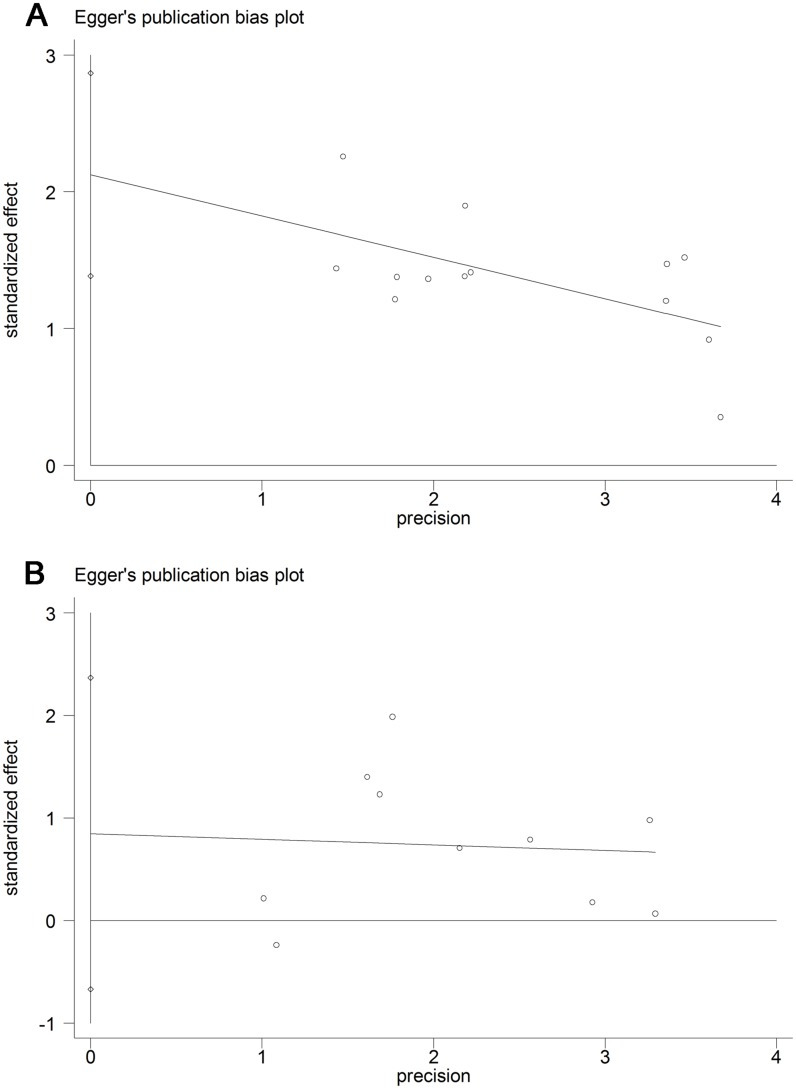
Egg’s test results of OS and RFS of PCa patients.

The funnel plot was only asymmetrical for OS. The *P* values of Begg’s and Egger’s tests for studies indicated the presence of publication bias in terms of OS (*P* = 0.001 and *P* < 0.001) but not RFS (*P* = 0.210 and *P* = 0.235) among the included studies (Figs 4 and 5).

Accordingly, we performed a “trim and fill” analysis for studies focusing on OS. Six studies evaluating the prognostic value of NLR in OS were estimated to be unpublished. The filled meta-analysis (HR 1.424, 95% CI 1.231–1.647, *P* < 0.001) was also similar to our pooled results.

### Sensitivity analysis

A sensitivity analysis was performed in which one study was deleted at a time (S5 Fig). The pooled HRs for OS and RFS were insignificantly changed, thereby suggesting the robustness of the results.

## Discussion

The present meta-analysis indicated that the inflammation marker NLR had prognostic values for OS and RFS in patients with PCa. Although high pretreatment NLR had a significant prognostic value in patients with advanced PCa, such NLR had no predictive value in terms of OS in patients with localized PCa. Therefore, higher pretreatment NLR had a stronger predictive effect in patients with more advanced disease. In terms of RFS, patients with higher pretreatment NLR had shorter RFS than those with lower NLR in both localized and advanced PCa; however, the association was more significant in patients with advanced PCa. Two previous studies in patients with colorectal and lung cancers also concluded that the prognostic value of NLR was higher in more advanced cancers [22, 23].

According to the subgroup analyses, NLR was significantly correlated with OR and RFS based on the multivariate analysis. Therefore, NLR was an independent risk factor for the outcomes. The further subgroup analysis showed that an NLR cut-off value ≥3 had a more significant prognostic value than a cut-off value <3 (supplemental data). Thus, a higher NLR cut-off may increase the specificity for predicting a poor prognosis in patients with PCa.

NLR was also associated with lymph node involvement but not with pathological stage or Gleason score. This deduction is consistent with the conclusion that NLR is an independent prognostic marker for PCa. A study that assessed NLR in esophageal cancer also showed that NLR was associated with tumor invasion and lymph node metastasis but not with tumor differentiation or vascular invasion [24]. In addition, NLR was associated with vascular invasion but not with tumor differentiation in hepatic cancer [25]. These observations suggest that inflammation severity may affect metastasis progression in PCa but not intrinsic tumor characteristics, such as tumor stage and Gleason score. The results of our analysis showed that the prognostic values of NLR in terms of OS or RFS were more significant in mCRPC, thereby supporting the association between inflammation and metastatic progression. Given the limited number of studies enrolled in this meta-analysis, these conclusions require further investigation.

Heterogeneity was observed in the included studies. This heterogeneity may be partially explained by geographic area, patient type, and sample size. Significant heterogeneity in selection bias is inevitable in studies with smaller sample sizes. Moreover, the treatments adopted in the studies also varied. Baseline pretreatment, types and doses of chemotherapy regimens, and dichotomized cut-off values also differed among the studies. However, the subgroup analysis showed that the prognostic value of NLR was unaffected by the factors included in the analysis. In addition, the sensitivity analysis indicated that our results were relatively conclusive. The results of the random-effect model were similar to those of the fixed-effect model, thus indicating that the pooled results were robust.

Therefore, NLR could serve as a prognostic marker because of the following reasons. First, inflammation is theoretically a critical component in the pathogenesis and progression of cancer [26]. Inflammatory mediators and pro-inflammatory cytokines are systemically elevated in patients with advanced cancer and contribute to the development of the acute phase response. NLR is an indicator of systemic inflammation and general immune response of the host [27–30]. It is also correlated with prognosis across a wide variety of tumor types [31–36]. Second, NLR can be available from blood routine test in daily clinical practice, an approach that is low cost, convenient, and reproducible. Finally, as shown in the present meta-analysis, NLR is closely associated with the progress and prognosis of advanced PCa patients. Therefore, NLR can better serve as a marker for advanced PCa patients.

NLR has other predictive values in PCa. Higher NLR was associated with lower prostate-specific antigen response to abiraterone or docetaxel chemotherapy [13, 37]. Thus, NLR could be used to estimate the probability of response to adjuvant therapy. In addition, NLR could also act as a biomarker to predict PCa in men undergoing prostate needle biopsy [38] and to predict the progression of benign prostatic hyperplasia [39]. However, NLR prior to prostate biopsy was higher in patients with prostatitis than in those with PCa or benign prostate hyperplasia [40]. Therefore, patients with prostatitis must be excluded when employing NLR as a biomarker for PCa.

This study has several limitations. First, most of the included studies were retrospective and thus more susceptible to bias in data selection and analysis. Second, the NLR cut-off values in the included studies ranged from 2 to 5, and this heterogeneity could hinder the application of these ratios in the clinical setting. Therefore, more original research is required to determine the most suitable NLR cut-off value. Third, one study investigating OS did not include NLR in the multivariate analysis because it failed to gain statistical significance in the univariate analysis. Thus, the corresponding HR and 95% CI could only be retrieved from univariate analysis. For BRFS and CRFS, similar conditions were observed in two and four studies, which may have impaired the accuracy of the pooled analysis. Fourth, the included studies did not report cancer-specific survival, an outcome important for cancer survival analysis. Fifth, NLR could be affected by other conditions, such as acute coronary syndromes, valvular heart diseases, renal diseases, liver diseases, hypertension, inflammatory diseases, infection, and some medications [41–43]. However, the inaccessibility of these clinical data means that we were unable to include these factors in the pooled analysis.

Despite the above limitations, our meta-analysis supports the values of pretreatment NLR for predicting recurrence and survival outcome in patients with locally advanced or metastatic PCa. Notably, NLR can only predict the recurrence of localized PCa. NLR can be easily obtained from routine blood tests and thus may supplement prostate-specific antigen, Gleason score, and magnetic resonance imaging for evaluating the condition of patients.

## Conclusions

NLR is a useful predictive marker in the progress and prognosis of patients with locally advanced or metastatic PCa. Patients with higher NLR are more likely to have poorer prognosis than those with lower NLR.

## Supporting Information

S1 Fig Meta-analysis of the NLR value and OS in the subgroup of PCa patients according to NLR value. (TIF)

S2 Fig Meta-analysis of the NLR value and RFS in the subgroup of PCa patients according to NLR value. (TIF)

S3 Fig Meta-analysis of the NLR value and OS in the subgroup of PCa patients according to patient type. (TIF)

S4 Fig Meta-analysis of the NLR value and RFS in the subgroup of PCa patients according to patient type. (TIF)

S5 Fig Sensitivity analysis for the pooled analysis of OS and RFS. (TIF)

S1 Table Checklist of items to include when reporting a systematic review or meta-analysis. (DOC)

S2 Table Characteristics of included studies. (DOC)

S3 Table Results of the association between NLR and overall survival. (DOC)

S4 Table Results of the association between NLR and recurrence-free survival. (DOC)

S5 Table Heterogeneity test and publication bias analyses among included studies. (DOC)

S6 Table Results of the meta-analysis on the association between NLR and clinical features in PCa. (DOC)
