# Supplementary figures and images for: Prognostic Value of Neutrophil-to-Lymphocyte Ratio in Localized and Advanced Prostate Cancer: A Systematic Review and Meta-Analysis
Source: PLoS One. 2016 Apr 20;11(4):e0153981. doi: 10.1371/journal.pone.0153981 (PMC4838250; doi:10.1371/journal.pone.0153981)

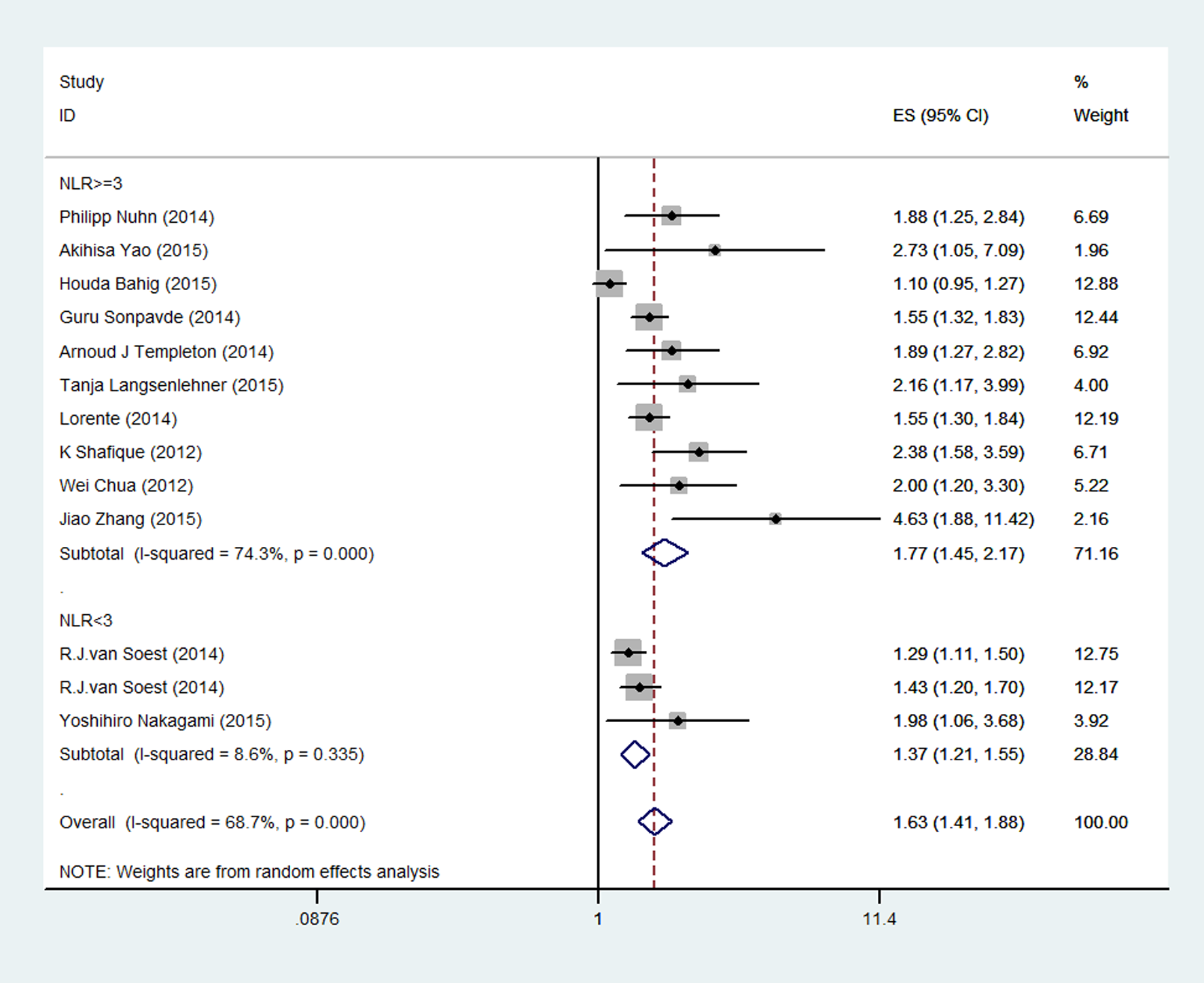

Supplement: S1 Fig — (TIF) [file pone.0153981.s001.tif]

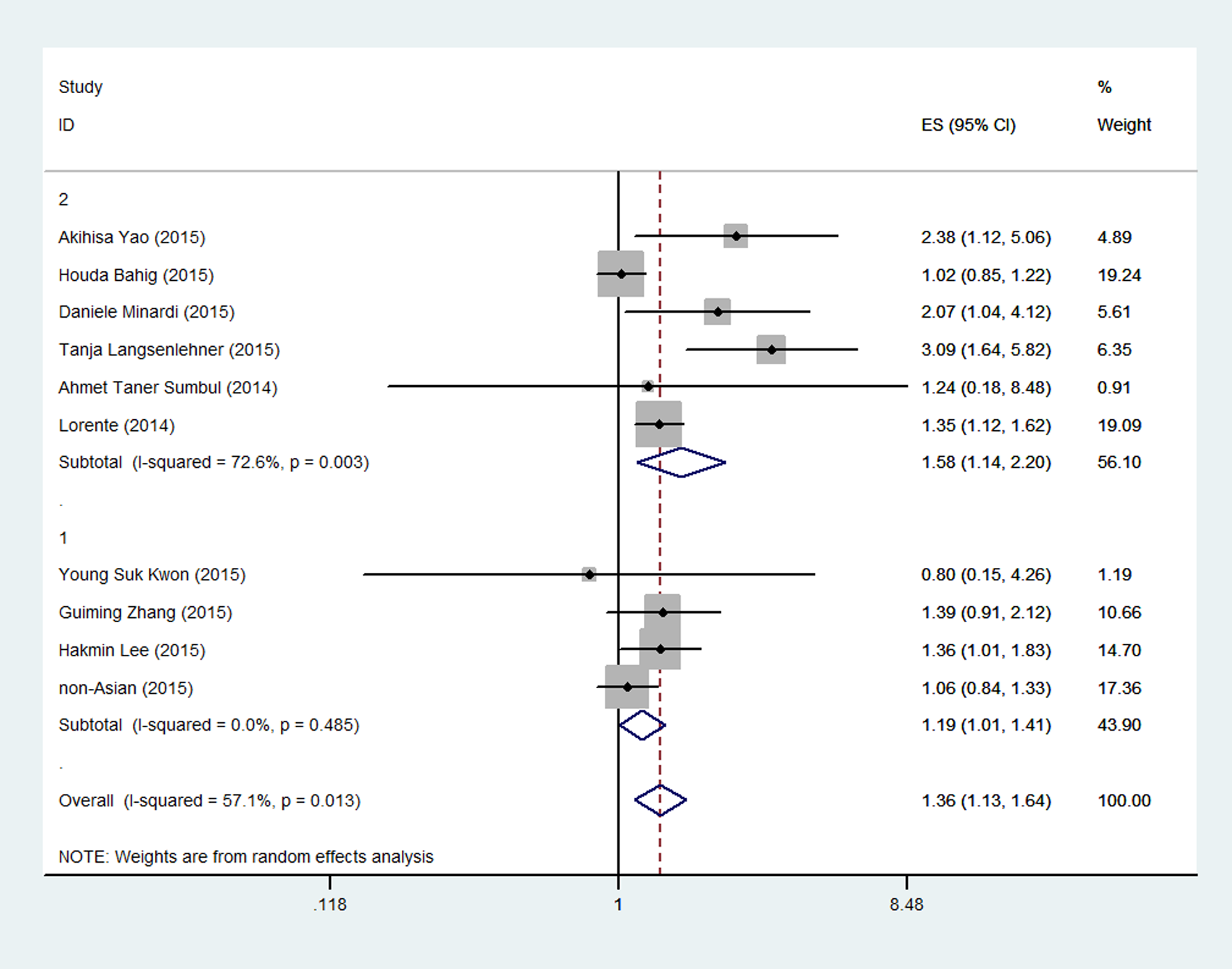

Supplement: S2 Fig — (TIF) [file pone.0153981.s002.tif]

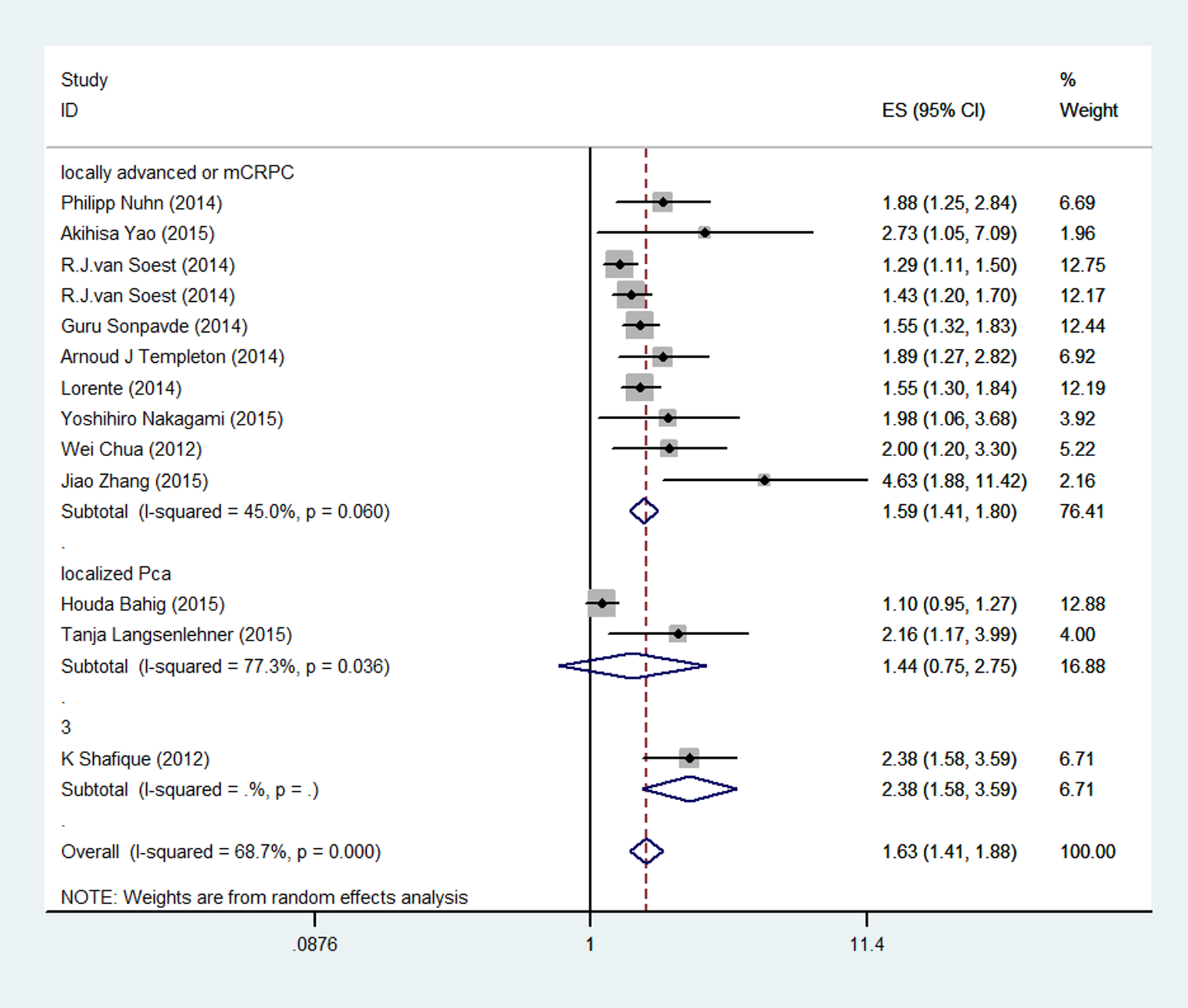

Supplement: S3 Fig — (TIF) [file pone.0153981.s003.tif]

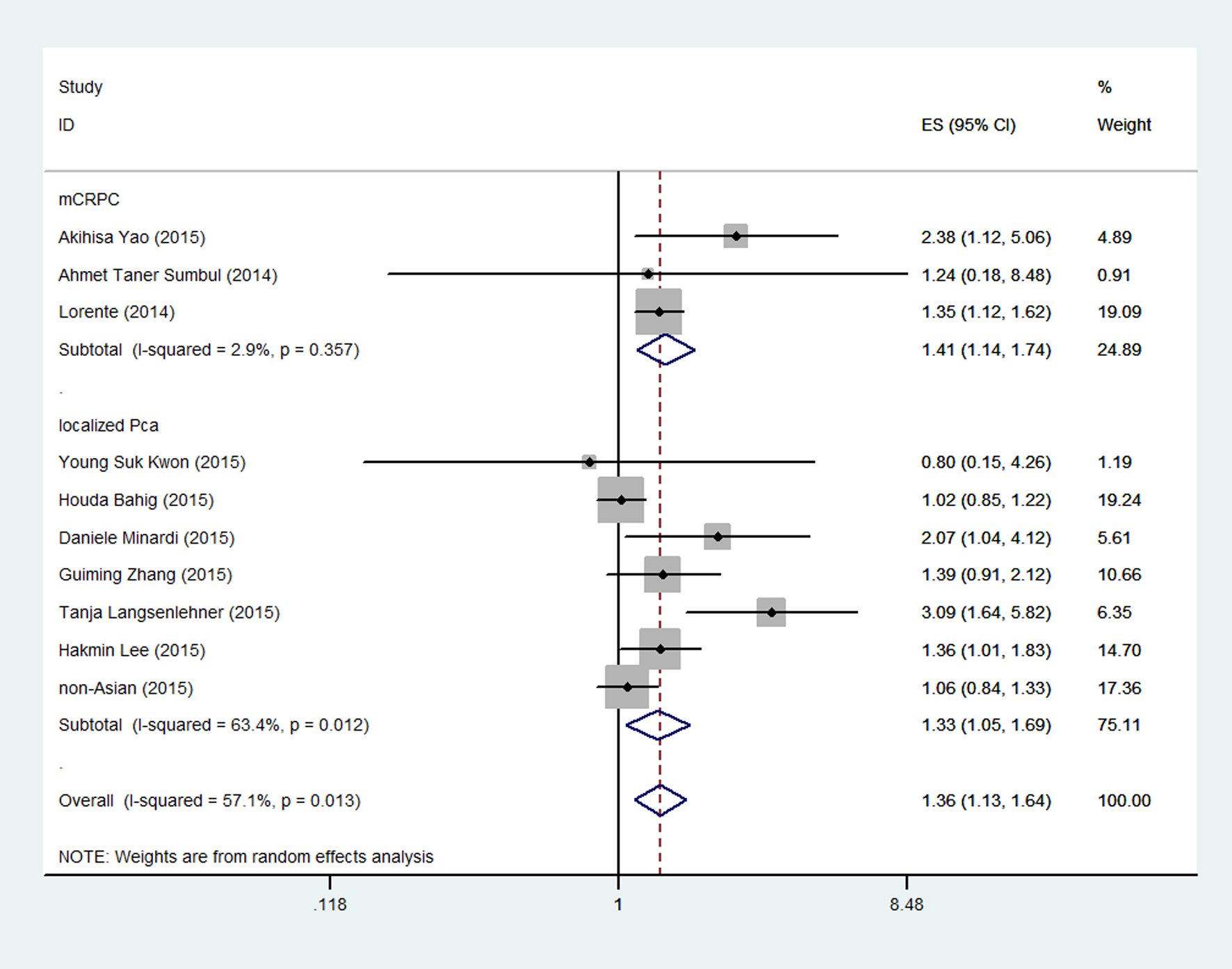

Supplement: S4 Fig — (TIF) [file pone.0153981.s004.tif]

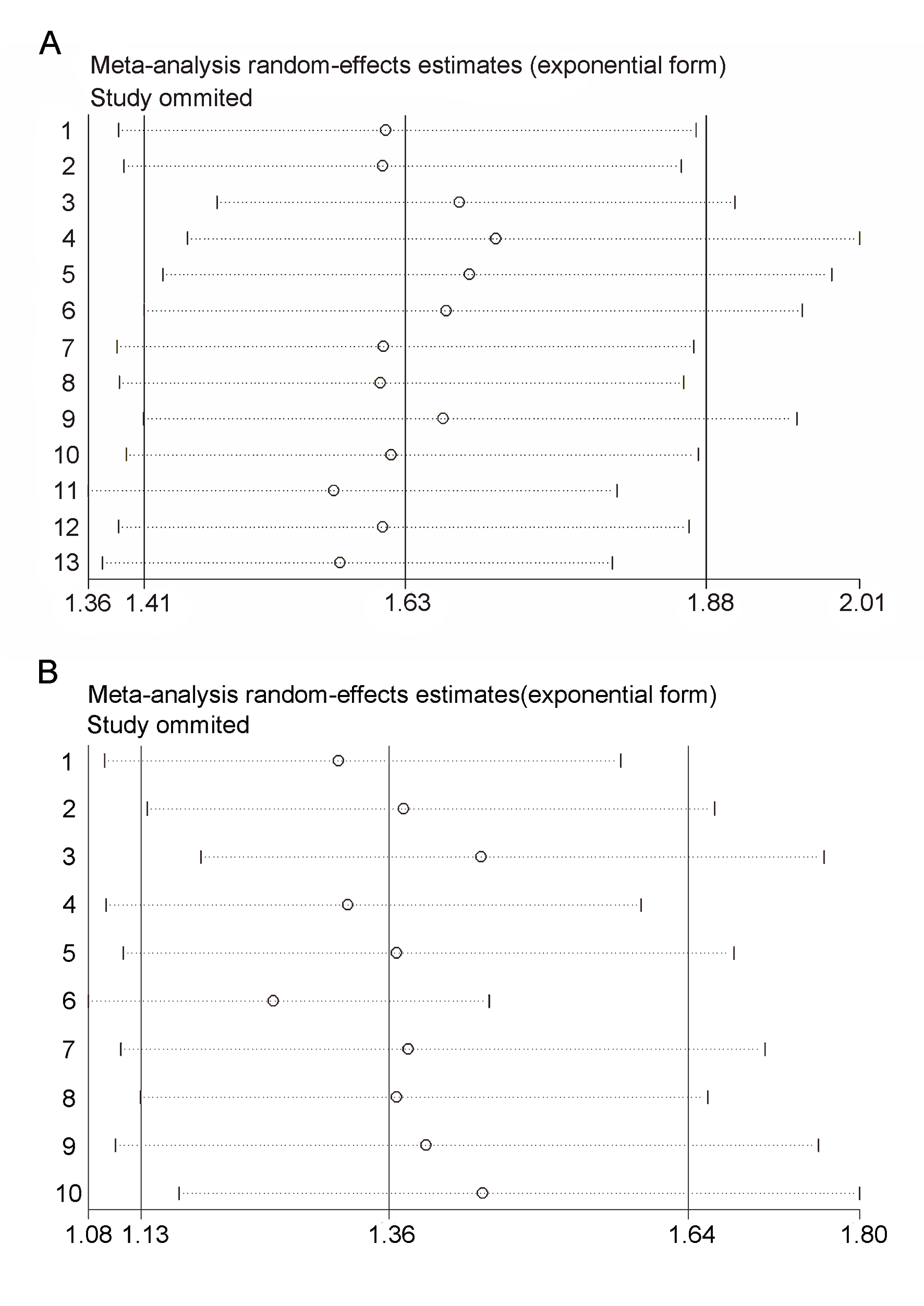

Supplement: S5 Fig — (TIF) [file pone.0153981.s005.tif]
