# Supplementary material for: Prognostic Value of Neutrophil-to-Lymphocyte Ratio in Localized and Advanced Prostate Cancer: A Systematic Review and Meta-Analysis
Source: PLoS One. 2016 Apr 20;11(4):e0153981. doi: 10.1371/journal.pone.0153981 (PMC4838250; doi:10.1371/journal.pone.0153981)
Supplement: S2 Table — (DOC) [file pone.0153981.s007.doc]

| **Supplemental table 2 Characteristics of included studies** | | | | | | | | | | | |
| --- | --- | --- | --- | --- | --- | --- | --- | --- | --- | --- | --- |
| **Study** | **Country** | **Year** | **Patients** | **N** | **Age,y** | **Treatment** | **Cut off** | **NLR H/L** | **Endpoint** | **Follow-up** | **NOS score** |
| **Philipp Nuhn** | USA | 2014 | mCRPC | 238 | NA | Docetaxel | 3 | 168/70 | OS | 15.0(1.5-90.2) | 7 |
| **Akihisa Yao** | Japan | 2015 | mCRPC | 57 | 74(55-91) | Docetaxel | 3.5 | 27/30 | OS, cRFS | NA | 8 |
| **Young Suk Kwon** | USA | 2015 | Localized PCa | 217 | 59 (55-63) | RARP | 2.6 | 107/110 | RFS(BCR) | 18 | 7 |
| **Houda Bahig** | Canada | 2015 | Localized PCa | 950 | 68(44-87) | Curative radiotherapy | 3 | 475/475 | OS, RFS(BCR) | 44(1-156) | 7 |
| **R.J.van Soest** | Netherland | 2014 | mCRPC | 1224 | 68(40-88） | Docetaxel | 2 | 612/612 | OS | NA | - |
| **R.J.van Soest** | Netherland | 2014 | mCRPC | 1006 | 68(36-92） | Docetaxel or mitoxantrone | 2.1 | 503/503 | OS | NA | - |
| **Daniele Minardi** | Italy | 2015 | Localized PCa | 389 | 65(42-77) | Radical prostatectomy | 3 | NA | cRFS | 51.5(9-108) | 7 |
| **Guiming Zhang** | China | 2015 | Localized PCa | 237 | 68(41-84） | Radical prostatectomy | 2.36 | 76/161 | RFS(BCR) | 46.6 | 8 |
| **Guru Sonpavde** | USA | 2014 | mCRPC | 848 | 68(39-90) | docetaxel | 5 | 235/613 | OS | NA | 7 |
| **Tanja Langsenlehner** | European Cohort | 2015 | Localized PCa | 415 | 68.2(69.9-7.2) | 3D conformal radiotherapy | 5 | 65/350 | OS,cRFS | 87 | 7 |
| **Hakmin Lee** | Korea | 2015 | Localized PCa | 1367 | 67(45-82) | Radical prostatectomy | 2.5 | 158/1209 | RFS(BCR) | 57(IQR:39-72) | 8 |
| **Ahmet Taner Sumbul** | Turkey | 2014 | mCRPC | 33 | Mean71.24+-7.34 | Docetaxel | 3 | 18/15 | cRFS | NA | 6 |
| **Lorente** | Multinational | 2014 | mCRPC | 755 | 67(IQR:62-73) | Cabazitaxel OR mitoxantrone | 3 | NA | OS,RFS(BCR) | 12.8(IQR:7.8-16.9) | 6 |
| **Yoshihiro Nakagami** | Japan | 2015 | CRPC | 101 | NA | NA | 2.6 | NA | OS | 18 | 7 |
| **K Shafique** | UK | 2012 | Localized PCa | 709 | NA | NA | 5 | 304/405 | OS | NA | 6 |
| **Wei Chua** | Australia | 2012 | Advanced or metastatic | 68 | 63(28-85) | Docetaxel | 5 | 38/30 | OS | <30months | 6 |
| **Jiao Zhang** | China | 2015 | CRPC | 48 | 70(50-88) | Docetaxel | 3 | 24/24 | OS | 48(36-60) | 8 |
| **Poyet, C** | Switzerland | 2015 | Localized PCa | 399 | 64(41-78) | Radical prostatectomy | 2.67 | NA | cRFS | 23(0-65) |  |

NLR, Neutrophil-to-lymphocyte ratio; NOS, Newcastle-ottawa quality assessment scale; OS, Overall survival; cRFS, Clinical reccurence-free survival; RFS(BCR), Reccurence-free survival (biochemical reccurence); mCRPC, metastatic castration-resistant prostate cancer; PCa, Prostate cancer
