# Supplementary material for: Prognostic Value of Neutrophil-to-Lymphocyte Ratio in Localized and Advanced Prostate Cancer: A Systematic Review and Meta-Analysis
Source: PLoS One. 2016 Apr 20;11(4):e0153981. doi: 10.1371/journal.pone.0153981 (PMC4838250; doi:10.1371/journal.pone.0153981)
Supplement: S3 Table — (DOC) [file pone.0153981.s008.doc]

**Supplemental table 3 Results of the association between NLR and overall survival**

| **Study** | **OS Univariate**  **HR** | **lCI** | **UCI** | **OS Multivariate HR** | **lCI** | **UCI** |
| --- | --- | --- | --- | --- | --- | --- |
| **Philipp Nuhn** | 1.675 | 1.219 | 2.302 | 1.883 | 1.248 | 2.843 |
| **Akihisa Yao** | 2.795 | 1.194 | 6.545 | 2.728 | 1.05 | 7.088 |
| **Houda Bahig** | 1.1 | 0.95 | 1.27 | NA | NA | NA |
| **R.J.van Soest(1)** | NA | NA | NA | 1.29 | 1.11 | 1.5 |
| **R.J.van Soest(2)** | NA | NA | NA | 1.43 | 1.2 | 1.7 |
| **Guru Sonpavde** | 2 | 1.72 | 2.32 | 1.55 | 1.32 | 1.83 |
| **Arnoud J Templeton** | 1.65 | 1.25 | 2.18 | 1.89 | 1.27 | 2.82 |
| **Tanja Langsenlehner** | 2.1 | 1.17 | 3.78 | 2.16 | 1.17 | 3.99 |
| **Lorente** | 1.55 | 1.3 | 1.84 | NA | NA | NA |
| **Yoshihiro Nakagami** | NA | NA | NA | 1.98 | 1.06 | 3.68 |
| **K Shafique** | 2.89 | 1.95 | 4.29 | 2.38 | 1.58 | 3.59 |
| **Wei Chua** | NA | NA | NA | 2 | 1.2 | 3.3 |
| **Jiao Zhang** | 2.69 | 1.64 | 4.4 | 4.625 | 1.875 | 11.418 |

NLR, neutrophil-to-lymphocyte ratio; OS, overall survival; LCI, lower confidence interval; UCI, upper confidence interval; HR, hazard ratio
