# Supplementary material for: Prognostic Value of Neutrophil-to-Lymphocyte Ratio in Localized and Advanced Prostate Cancer: A Systematic Review and Meta-Analysis
Source: PLoS One. 2016 Apr 20;11(4):e0153981. doi: 10.1371/journal.pone.0153981 (PMC4838250; doi:10.1371/journal.pone.0153981)
Supplement: S4 Table — (DOC) [file pone.0153981.s009.doc]

**Supplemental table 4 Results of the association between NLR and recurrence-free survival**

| **Study** | **RFS(BCR) Uni. HR** | **LCI** | **UCI** | **RFS(BCR) Multi.**  **HR** | **ICI** | **95% UCI** | **cRFS**  **Uni.**  **HR** | **LCI** | **UCI** | **cRFS**  **Multi.**  **HR** | **LCI** | **UCI** |
| --- | --- | --- | --- | --- | --- | --- | --- | --- | --- | --- | --- | --- |
| **Akihisa Yao** | NA | NA | NA | NA | NA | NA | 2.337 | 0.479 | 3.785 | 2.376 | 1.123 | 5.056 |
| **Young Suk Kwon** | 0.8 | 0.15 | 4.26 | NA | NA | NA | NA | NA | NA | NA | NA | NA |
| **Daniele Minardi** | NA | NA | NA | NA | NA | NA | 2.07 | 1.04 | 4.12 | NA | NA | NA |
| **Guiming Zhang** | 1.79 | 1.22 | 2.63 | 1.388 | 0.909 | 2.118 | NA | NA | NA | NA | NA | NA |
| **Tanja Langsenlehner** | NA | NA | NA | NA | NA | NA | 2.01 | 1.1 | 3.67 | 3.09 | 1.64 | 5.82 |
| **Hakmin Lee** | 2.547 | 1.969 | 3.294 | 1.358 | 1.008 | 1.829 | NA | NA | NA | NA | NA | NA |
| **Ahmet Taner Sumbul** | NA | NA | NA | NA | NA | NA | 1.24 | 0.18 | 8.48 | NA | NA | NA |
| **Lorente** | 1.35 | 1.12 | 1.62 | NA | NA | NA | NA | NA | NA | NA | NA | NA |
| **Poyet, C** | NA | NA | NA | NA | NA | NA | 1.061 | 0.844 | 1.334 | NA | NA | NA |

NLR, neutrophil-to-lymphocyte ratio; Uni. Univariate; Multi. Multivariate; cRFS, Clinical reccurence-free survival; RFS(BCR), Reccurence-free survival (biochemical reccurence); LCI, lower confidence interval; UCI, upper confidence interval; HR, hazard ratio
