# Supplementary material for: Prognostic Value of Neutrophil-to-Lymphocyte Ratio in Localized and Advanced Prostate Cancer: A Systematic Review and Meta-Analysis
Source: PLoS One. 2016 Apr 20;11(4):e0153981. doi: 10.1371/journal.pone.0153981 (PMC4838250; doi:10.1371/journal.pone.0153981)
Supplement: S5 Table — (DOC) [file pone.0153981.s010.doc]

|  | **Overall survival** | | | | **Recurrence free survival** | | | |
| --- | --- | --- | --- | --- | --- | --- | --- | --- |
|  | Heterogeneity | | Publication bias | | Heterogeneity | | Publication bias | |
|  | P^a^ | I2(%)^b^ | P^c^ | P^d^ | P^a^ | I2(%)^b^ | P^c^ | P^d^ |
| **Overall** | 0 | 68.7 | 0.001 | 0 | 0.013 | 57.1 | 0.21 | 0.235 |
| **Geographic area** |  |  |  |  |  |  |  |  |
| 1.Asian | 0.492 | 0 | 0.734 | 0.414 | 0.391 | 0 | 0.296 | 0.228 |
| 2.non-Asian | 0.004 | 64.6 | 0.005 | 0.004 | 0.009 | 64.7 | 0.548 | 0.479 |
| **statistical methods** |  |  |  |  |  |  |  |  |
| 1.univariate | 0 | 49.77 | 0.076 | 0.047 | 0 | 79.4 | 0.283 | 0.515 |
| 2.multivariate | 0.014 | 22.16 | 0.008 | 0 | 0.079 | 55.8 | 0.308 | 0.129 |
| **Patient** |  |  |  |  |  |  |  |  |
| 1.Localized | 0.036 | 77.3 | 0.296 | 0.171 | 0.012 | 63.4 | 0.133 | 0.289 |
| 2.Advanced | 0.06 | 45 | 0.004 | 0.001 | 0.357 | 2.9 | 1 | 0.738 |
| **sample size** |  |  |  |  |  |  |  |  |
| 1.<800 | 0.248 | 21.9 | 0.048 | 0.006 | 0.038 | 52.8 | 0.711 | 0.474 |
| 2.>=800 | 0.014 | 71.8 | 0.308 | 0.179 | 0.107 | 61.4 | 1 | NA |
| **NLR standard** |  |  |  |  |  |  |  |  |
| 1.<3 | 0.335 | 8.6 | 0.296 | 0.171 | 0.485 | 0 | 1 | 0.653 |
| 2.>=3 | 0.000 | 74.3 | 0.004 | 0.001 | 0.003 | 72.6 | 0.452 | 0.172 |
| **follow-up** |  |  |  |  |  |  |  |  |
| 1.<18 | 0.56 | 0 | 0.296 | 0.11 | NA | NA | NA | NA |
| 2.>=18 | 0.001 | 82.7 | 0.06 | 0.011 | 0.012 | 63.4 | 0.133 | 0.289 |
| **Recurrence type** |  |  |  |  |  |  |  |  |
| 1.BCR | NA | NA | NA | NA | 0.199 | 33.3 | 0.806 | 0.716 |
| 2.clinical recurrence | NA | NA | NA | NA | 0.006 | 72.3 | 1 | 0.327 |
|  |  |  |  |  |  |  |  |  |
| **pathological stage** | 0 | 93.8 | 0.296 | 0.265 |  |  |  |  |
| **Gleason score** | 0 | 83.8 | 0.734 | 0.168 |  |  |  |  |
| **lymphnode involvement** | 0.688 | 0 | NA | NA |  |  |  |  |

**Supplemental table 5 Heterogeneity test and publication bias analyses among included studies**

a P for heterogeneity within each subgroup.
b Proportion of between-study heterogeneity accounting for total heterogeneity.
c P values of Begg’s test.
d P values of Egger’s test.
