# Supplementary material for: Prognostic Value of Neutrophil-to-Lymphocyte Ratio in Localized and Advanced Prostate Cancer: A Systematic Review and Meta-Analysis
Source: PLoS One. 2016 Apr 20;11(4):e0153981. doi: 10.1371/journal.pone.0153981 (PMC4838250; doi:10.1371/journal.pone.0153981)
Supplement: S6 Table — (DOC) [file pone.0153981.s011.doc]

|  | n | OR | LCI | UCI | Heterogeneity | | Publication bias | |
| --- | --- | --- | --- | --- | --- | --- | --- | --- |
|  |  |  |  |  | Pa | I2(%) | Pc(Begg's test) | Pd(Egger'test) |
| **pathological stage**  T1-2 vs.T3-4 | 3 | 0.827 | 0.637 | 1.074 | 0 | 93.8 | 0.296 | 0.265 |
| **Reccurence free survival**  <7 vs >=7 | 4 | 0.761 | 0.555 | 1.044 | 0 | 83.8 | 0.734 | 0.168 |
| **lymphnode involvement**  YES vs. NO | 2 | 1.616 | 1.167 | 2.239 | 0.688 | 0 | NA | NA |

**Supplemental table 6 Results of the meta-analysis on the association between NLR and clinical features in PCa**

NLR, Neutrophil-to-lymphocyte ratio; OR odds ratio; LCI, lower confidence interval; UCI, upper confidence interval
